# Internet Users’ Valuation of Enhanced Data Protection on Social Media: Which Aspects of Privacy Are Worth the Most?

**DOI:** 10.3389/fpsyg.2018.01516

**Published:** 2018-08-22

**Authors:** Jasmin Mahmoodi, Jitka Čurdová, Christoph Henking, Marvin Kunz, Karla Matić, Peter Mohr, Maja Vovko

**Affiliations:** ^1^Swiss Center for Affective Sciences, University of Geneva, Geneva, Switzerland; ^2^Department of Psychology, Masaryk University, Brno, Czechia; ^3^Department of Psychological and Behavioural Science, London School of Economics and Political Science, London, United Kingdom; ^4^Faculty of Social and Behavioral Science, University of Groningen, Groningen, Netherlands; ^5^Department of Psychology, University of Leuven, Leuven, Belgium; ^6^Department of Psychology, University of Amsterdam, Amsterdam, Netherlands; ^7^Department of Psychology, University of Ljubljana, Ljubljana, Slovenia

**Keywords:** information privacy, privacy concerns, willingness-to-pay, social networking services, Facebook, premium products, privacy dimensions

## Abstract

As the development of the Internet and social media has led to pervasive data collection and usage practices, consumers’ privacy concerns have increasingly grown stronger. While previous research has investigated consumer valuation of personal data and privacy, only few studies have investigated valuation of different privacy aspects (e.g., third party sharing). Addressing this research gap in the literature, the present study explores Internet users’ valuations of three different privacy aspects on a social networking service (i.e., Facebook), which are commonly captured in privacy policies (i.e., data collection, data control, and third party sharing). A total of 350 participants will be recruited for an experimental online study. The experimental design will consecutively contrast a conventional, free-of-charge version of Facebook with four hypothetical, privacy-enhanced premium versions of the same service. The privacy-enhanced premium versions will offer (1) restricted data collection on side of the company; (2) enhanced data control for users; and (3) no third party sharing, respectively. A fourth premium version offers full protection of all three privacy aspects. Participants’ valuation of the privacy aspects captured in the premium versions will be quantified measuring willingness-to-pay. Additionally, a psychological test battery will be employed to examine the psychological mechanisms (e.g., privacy concerns, trust, and risk perceptions) underlying the valuation of privacy. Overall, this study will offer insights into valuation of different privacy aspects, thus providing valuable suggestions for economically sustainable privacy enhancements and alternative business models that are beneficial to consumers, businesses, practitioners, and policymakers, alike.

## Introduction

The advent of the Internet and social media has drastically transformed all aspects of our lives; how we work, consume, and communicate (see also Stewart and Segars, 2002; Paine et al., 2007). While this has had considerable advantages for society overall, the growing influence of the Internet and technologies has always been linked to concerns for privacy and the collection and use of personal information (e.g., Zuboff, 1988). The threats to individual privacy through these technologies have been repeatedly documented. Over the past years, sensitive personal data were repeatedly unlawfully obtained and mishandled in numerous data breaches. Most recently, sensitive personal information, including credit scores, of almost 150 million people was compromised in the 2017 Equifax data breach (e.g., Zou and Schaub, 2018) and around 87 million Facebook users were impacted by the Cambridge Analytica data scandal in 2018 (e.g., Revell, 2018).

While some consumers are unaware of the data they produce or of the full extent to which their data are mined and analyzed (e.g., Turow et al., 2005), others do not care (Garg et al., 2014). A majority of consumers, however, report concerns about their online privacy (e.g., Phelps et al., 2000; Pew Research Center, 2014), and, yet, most people often trade their personal data for online services and products (Carrascal et al., 2013). For instance, even privacy-concerned individuals join social networking services, such as Facebook, and share large amounts of personal information on these platforms (Acquisti and Gross, 2006).

Several factors play a role in explaining the discrepancy between people’s concerns and their online sharing behaviors, such as bounded rationality, cognitive biases and heuristics, or social factors (see Kokolakis, 2017 for a review). One explanation is the so-called privacy calculus, which postulates that people perform a calculus of the costs (i.e., loss of privacy) and benefits (i.e., gain from information disclosure). Their final decisions and behaviors are a result of this calculus and determined by the outcome of this trade-off. When the perceived benefits outweigh the perceived costs, people are likely to disclose information (Culnan and Armstrong, 1999; Dinev and Hart, 2006b). Other factors accounting for this discrepancy are, for instance, that privacy functionalities are often not usable leaving users with little choice or alternatives and making it almost impossible for users to act upon their concerns (Iachello and Hong, 2007; Lipford et al., 2008). Experts call for better data and privacy regulations as well as alternative business models to balance the asymmetric relationship between consumers and business (e.g., Zuckerman, 2014; Tufekci, 2015; Gasser, 2016; New York Times, 2018; Quito, 2018). Understanding Internet users’ privacy concerns and valuations is essential to develop strategies that match users’ needs and enable them to act in accordance to their concerns.

The present research investigates Internet users’ concerns and valuation of privacy in the context of the social networking service Facebook. In the experimental online study, participants will be presented premium versions of Facebook that offer different privacy enhancements (e.g., less data collection, more data control, and no third party sharing) for a monthly fee. Participants will be asked to indicate their willingness-to-pay for these privacy enhancements. In addition, psychological mechanisms underlying these valuations will be examined. In the following, the scientific literature underlying this research will be reviewed and the research hypotheses for this research will be developed. The experimental design and research methods will be outlined and the anticipated results presented and discussed.

## Theoretical Background

Privacy concerns have become one of the most central themes in the digital era, likewise for scholars, consumers, businesses, practitioners, and policy-makers. Acquisti and Gross (2009), for example, demonstrated the threat to individual privacy by inferring identities (i.e., social security numbers) through supposedly “anonymized” data. Other research showed that sensitive personal information, such as sexual orientation, could be inferred from Facebook Likes and facial images (Kosinski et al., 2013; Wang and Kosinski, 2018). Most recently, several data breaches, such as the Cambridge Analytica scandal that compromised personal data of about 87 million Facebook users worldwide (Revell, 2018), have sparked ethical debates on users’ online privacy (e.g., Zunger, 2018).

Although not a novel concept, there is no clear consensus on the definition of privacy (Solove, 2006). Privacy is a complex, multidimensional construct that has been studied from different perspectives (Laufer and Wolfe, 1977) and, accordingly, has been operationalized in many different ways (e.g., as an attitude in Buchanan et al., 2007; as a value in Earp et al., 2005; Alashoor et al., 2015; as a behavior in Jensen et al., 2005; or as a right in McCloskey, 1980; Warren and Brandeis, 1890; see also Bélanger and Crossler, 2011 for a review). In order to tackle privacy in a standardized and reliable manner, most contemporary research concerned with online privacy uses the construct of privacy concerns as a proxy to explore information privacy (see Dinev et al., 2009; Smith et al., 2011). Hence, a control-centered definition of information privacy prevails, where privacy is defined as individual ability to control disclosure and use of personal information (Westin, 1968; Altman et al., 1974; Margulis, 1977). Accordingly, privacy concerns can be defined as consumers’ perceptions of how the information they provide online will be used (Dinev and Hart, 2006a), and if this use can be regarded as ‘fair’ (Malhotra et al., 2004). Two widely accepted models of privacy exist that treat privacy concern as a multidimensional construct: The multidimensional instrument developed by Smith et al. (1996) assesses “individuals’ concerns about organizational information privacy practices” (p. 167). This instrument has been adapted by Malhotra et al. (2004), making it applicable to the context of online privacy. The Internet User’s Information Privacy Concerns (IUIPC) model consists of three dimensions, namely collection, control, and awareness. The dimension collection refers to users’ concerns regarding the collection of their personal information. The dimension control refers to users’ beliefs to have the right to determine and control how their information are collected, stored, and shared. The dimension awareness refers to users’ awareness of data privacy practices of companies (i.e., online service providers).

Despite the importance of privacy in the digital era, people – paradoxically even those holding strong privacy concerns – often trade their personal data for online services and products (Carrascal et al., 2013). For example, Acquisti and Gross (2006) demonstrated that even privacy-concerned individuals join the social networking service Facebook disregarding its privacy policies and revealing large amounts of personal information. The term “privacy paradox” has been coined to describe this dichotomy between expressed privacy concerns and actual online disclosure and sharing behaviors (Norberg et al., 2007). This paradox is particularly pronounced on social networking platforms, given the seemingly contradictory relationship between information privacy and social networking (i.e., connecting and sharing personal information with an online network; Lipford et al., 2012).

Many researchers have attempted to unravel and explain the privacy paradox (e.g., Barnes, 2006; Pötzsch, 2008; Sundar et al., 2013; Motiwalla and Li, 2016). One explanation defines the privacy paradox in terms of trade-offs between the benefits of using digital products and services and disclosing information online at the cost of a (partial) loss of privacy. These cost-benefit analyses are modeled as privacy calculus (Culnan and Armstrong, 1999), where privacy and personal information are conceptualized in economic terms as commodities (Klopfer and Rubenstein, 1977; Bennett et al., 1995). Willingness-to-pay is a commonly used indicator to quantify consumers’ economic valuation of commodities, such as goods and services (e.g., Casidy and Wymer, 2016; Lee and Heo, 2016). Accordingly, many scholars use willingness-to-pay as an indicator for economic valuations of privacy and information disclosure (e.g., Grossklags and Acquisti, 2007; Beresford et al., 2012; Spiekermann et al., 2012; Acquisti et al., 2013; Schreiner and Hess, 2015). Tsai et al. (2011) demonstrated that, when sufficient privacy information is available, people are willing to pay a premium to be able to purchase from websites that offer greater privacy protection. Studying low-priced products, the authors found that people were willing to pay up to 4% – around US$0.60 – more for enhanced privacy. Egelman et al. (2009) showed that people are willing to pay up to US$0.75 for increased privacy when online shopping, particularly when shopping for privacy-sensitive items. Similarly, a quarter of smartphone users were willing to pay a US$1.50 premium to use a mobile app that made fewer requests to access users’ personal information (Egelman et al., 2013). In a study by Hann et al. (2007) among U.S. Americans, personal information was worth US$30.49 – US$44.62. In another study, participants expressed high sensitivity to and concern for privacy, but only half of the participants were actually willing to pay for a change in data protection laws that would give them property rights to their personal data. The economic value placed on these privacy rights averaged around US$38 (Rose, 2005). Schreiner et al. (2013) tested privacy-enhanced premium versions of Facebook and Google and measured consumers’ propensity to pay for these services. The authors found that the optimal price for Facebook was aaa1.67/month and the optimal price for Google’s search engine lay between aaa1.00 and aaa1.50/month. Even though participants in the study were willing to pay for privacy-enhanced premium version, these valuations are relatively low (see also Bauer et al., 2012). Different explanations can account for the rather low valuations of privacy and data protection. For example, individuals who have not experienced invasion of their information privacy (e.g., through breaches or hacks) do not understand all the possible consequences resulting from information privacy violations and, therefore, tend to undervalue privacy (Hann et al., 2002). It might also be because many costs associated with the invasion of privacy occur from secondary use of information (Laudon, 1996), of which the consequences are often only experienced ex post (Acquisti, 2004). What is more, not all the costs of unprotected personal information are easy to quantify – while some of the consequences are tangible (e.g., identity theft), others are intangible (e.g., revealing personal life history to strangers; Brandimarte et al., 2015). Hence, it seems likely that people value privacy aspects that are tangible and immediate more than others.

In addition to these factors, several psychological characteristics have been identified in explaining consumers’ concerns and valuation of privacy. A large body of the literature shows that cognitive biases and heuristics, such as comparative optimism, overconfidence, or affect bias play an important role (see Kokolakis, 2017 for a review). For example, low privacy valuations are associated with people’s underestimation of one’s own and overestimation of other’s likelihood of experiencing misuse of personal data (Syverson, 2003; Baek et al., 2014), which could translate into low privacy valuations. Valuation of online privacy has also been linked to perceptions of usefulness, risk, and trust toward companies or services (e.g., Malhotra et al., 2004; Milne and Culnan, 2004; Dinev and Hart, 2006a; Garg et al., 2014; Schreiner and Hess, 2015). Prior context-specific disclosure behaviors are additional indicators of consumers’ valuations (Motiwalla et al., 2014). Therefore, it seems that the willingness-to-pay for online privacy is a telling measure, but only if considered in light of its psychological drivers.

While there is no shortage of willingness-to-pay studies trying to quantify the valuation of privacy (see also Acquisti et al., 2013), only very few studies have investigated the perception or valuation of different aspects of privacy. Hann et al. (2002) used conjoint analysis to examine the importance people ascribe to the different privacy concern dimensions of Smith et al. (1996), showing that websites’ secondary use of personal information is perceived as most important, followed by improper access of personal information. An earlier study using consumer ratings yielded similar results showing that consumers were more concerned about improper access and unauthorized secondary use than about data collection and possible errors in their data (Esrock and Ferre, 1999). Another conjoint analysis identified consumer segments based on their differing levels of privacy concerns, highlighting the need for different premium accounts that cater to consumers’ differing privacy preferences (Krasnova et al., 2009).

To our knowledge, no study has so far investigated whether these patterns can be replicated for Malhotra et al.’s (2004) adapted model of privacy concerns and no study has investigated consumers’ valuation of these privacy aspects in the context of social networking services. For example, a study by Schreiner et al. (2013) examined social media users’ willingness-to-pay for information privacy on Facebook, but did not differentiate between the three dimensions of privacy and, therefore, does not provide insights into which aspects of privacy are most valued by users. Additionally, the study by Schreiner and colleagues was limited in that they excluded non-members of Facebook, which constitutes an interesting consumer segment when it comes to privacy-enhanced premium versions of social networking services, as this segment may be especially interested in joining privacy-enhanced versions of such platforms.

## Study Objectives and Research Hypotheses

Filling these research gaps, the overarching objectives of the present study are twofold: first, the study will explore users’ valuation of three different privacy aspects in the context of social networking services and, second, the study will investigate the psychological mechanisms underlying users’ overall valuation of privacy.

Investigating the former, three privacy aspects will be studied that are captured in Facebook’s Data Policy (Facebook Inc., 2016) as well as in Malhotra et al.’s (2004) multidimensional model of privacy. These three privacy aspects are (1) data collection, (2) data control, and (3) third party use. Accordingly, participants will be offered enhancement of these three privacy aspects within hypothetical premium versions of Facebook. Precisely, these privacy-enhanced premium versions of Facebook will offer (1) restricted data collection on side of the company, (2) enhanced data control for users, and (3) no sharing of users’ data with third parties. Willingness-to-pay for the premium versions will be used as a proxy for participants’ valuation of these privacy aspects. Expanding on previous studies (e.g., Schreiner et al., 2013), this study’s insights will provide a more detailed understanding of users’ valuation of different aspects of privacy. It is explored whether Internet users value some aspects of privacy more than others. Though previous research suggests that third-party sharing may be valued most (Esrock and Ferre, 1999; Hann et al., 2002), we argue that it is also possible that companies’ restrictions on data collection may be valued more, since if no data are collected, users may be less worried about their data being shared with third parties. At the same time, the prevailing control-centered definition of privacy may invoke stronger valuations of the data control aspect. In light of these contradictory assumptions, for the present research no directional hypotheses can be formulated for the valuation of the three privacy aspects.

Investigating the latter, that is, the psychological mechanisms underlying valuation of privacy on social networking services, the present study will test a theoretical model that is developed and adapted based on proposed models by Malhotra et al. (2004) and Wilson and Valacich (2012). These models propose that privacy concerns increase perceived risk of information disclosure online and, thus, influence people’s intentions to protect their data. This relationship is expected to be further moderated by several other psychological and socio-demographic characteristics measured in this study. It is hypothesized that the proposed model will explain the psychological mechanisms underlying valuation of privacy on Facebook (see Section Theoretical Model).

## Materials and Methods

### Participants

We aim to recruit at least 350 English-speaking adults (i.e., minimum age of 18 years). The estimated sample size is based on Lipovetsky’s (2006) estimation that a minimum of 256 participants are needed to set up a price model with the precision of 𝜀 = 0.05 and to reach value close to 80%. Taking into account potential dropouts and invalid participant responses, we aim to reach sample size of a minimum of 350 participants. Though participant recruitment is restricted to English-speaking adults, we will, unlike previous studies (e.g., Schreiner et al., 2013), recruit participants across different countries^1^. As statistics report differing levels of privacy concerns and social media use across countries and cultures (e.g., Eurobarometer, 2016), we hope that our recruitment strategy will enable us to capture a heterogeneous participant sample with respect to the level of concern for and valuation of privacy. Furthermore, we will include both Facebook members and non-members in the sample. Facebook non-members are an important subsample, as this consumer segment could have a particular interest in privacy-enhanced versions of social networking services like Facebook. To ensure these sampling criteria, we will make use of various online channels, such as social networks and specialized study recruitment pages (e.g., findparticipants.com), as well as mailing lists, university platforms, and topic-relevant online forums.

### Experimental Design

**FIGURE 1:**
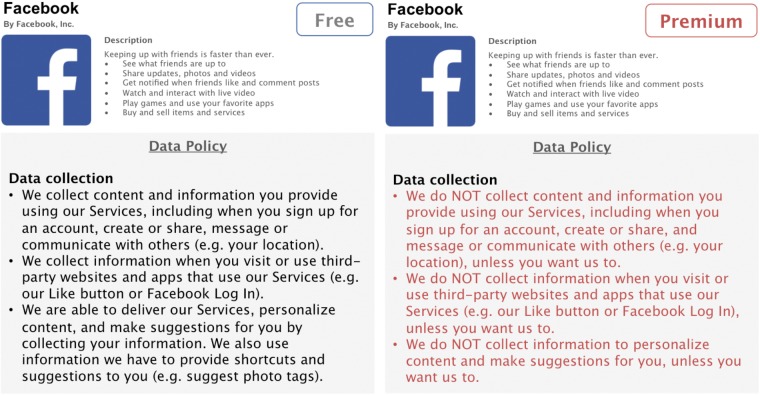
Condition 1: Enhanced *data collection*.

**FIGURE 2:**
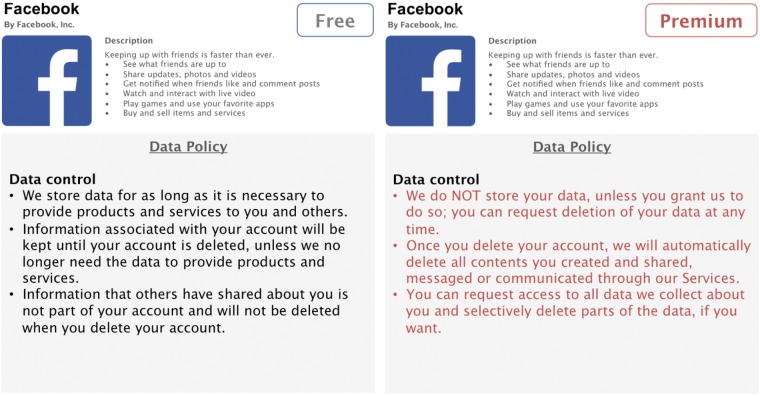
Condition 2: Enhanced *data control*.

**FIGURE 3:**
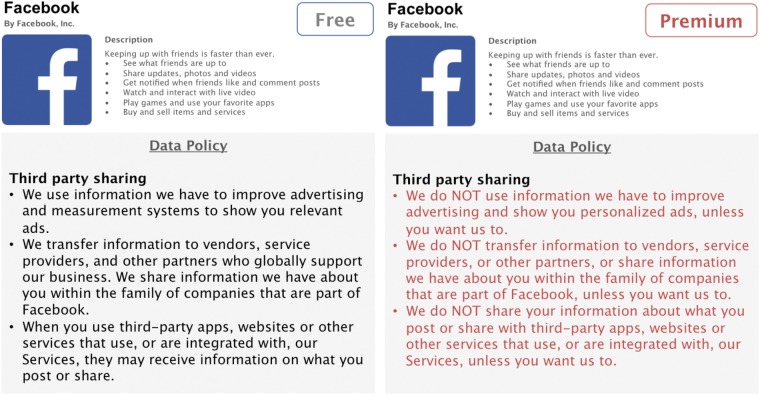
Condition 3: Enhanced *third party sharing*.

In the present online study, we will create four hypothetical privacy-enhanced premium versions of Facebook. The privacy enhancements of the premium versions will be based on three privacy aspects that are captured both in the IUIPC model (Malhotra et al., 2004) as well as in Facebook’s Data Policy (Facebook Inc., 2016). Three of these premium versions will have one specific privacy aspect enhanced: in the first condition, data collection policies will be less permissible, thus, granting users the option that Facebook collects less data about them; the second condition will offer enhanced data control for users and allows complete or selective deletion of stored data; in the third condition, users will have the option to opt out from having Facebook share their data with third parties, such as advertisers (see **Figures 1**–**3**). An additional fourth condition will consist all three privacy enhancements in a full-design premium version.

Designing these hypothetical premium versions as realistic as possible, we will rely on Facebook’s Data Policy to extract three central privacy aspects, namely data collection, data control, and third party sharing (Facebook Inc., 2016). We will adapt relevant parts of the policy accordingly to match the increased privacy functionalities of our premium versions. The conventional, free version of Facebook used for side-by-side comparisons consists of shortened and simplified, but otherwise unaltered, parts of Facebook’s original Data Policy. The premium versions are written in such a fashion to correspond to the original policy as much as possible, while enhancing specific privacy aspects. To facilitate readability, this information is presented in form of concise and comprehensive bullet points.

### Willingness-to-Pay Measure

Quantifying Internet users’ valuation of the different privacy aspects, the van Westendorp’s (1976) Price Sensitivity Meter model (PSM) will be employed as a willingness-to-pay measure. The PSM is a descriptive statistical procedure labeled the “psychological price” modeling (Lipovetsky et al., 2011). Rather than asking a single price indicator, the PSM allows capturing economic valuation in psychological terms. Furthermore, it ensures comparability of the results with the study by Schreiner et al. (2013). The PSM consists of four questions that ask participants to balance the value of certain products or services against the price. Precisely, participants will answer the following questions about the four premium versions (as compared to the free version) presented:

(1) At what price does this product become *too cheap*, that is, so cheap that you would question its quality and not buy it?
(2) At what price does this product start to seem *cheap* to you, that is, when does it start to seem like a bargain?
(3) At what price does this product start to seem *expensive* to you?
(4) At what price does this product become *too expensive*, that you would not consider buying it?

The questions will be presented simultaneously and in the above order below the two versions of Facebook (i.e., conventional, free-of-charge versus hypothetical, privacy-enhanced version of Facebook). Participants will be asked to indicate a monthly price they are willing to pay for the privacy enhancement of each premium version. Combining the answers from the four PSM questions will allow identifying the upper and the lower price limit that participants are willing to pay for privacy. Based on this, the optimal price can be calculated as described in more detail in Section Proposed Analysis.

After answering the four PSM questions, a single-item willingness-to-pay measure will be employed to additionally assess the overall willingness-to-pay for the different privacy aspects (“*Overall, how much would be willing to pay for this premium version of Facebook?”)*. This overall valuation measure will be used to validate the results of the PSM and to conduct the multiple comparisons between the three privacy enhancements, which will allow drawing conclusions about which privacy aspects are valued the most.

### Theoretical Model

**FIGURE 4:**
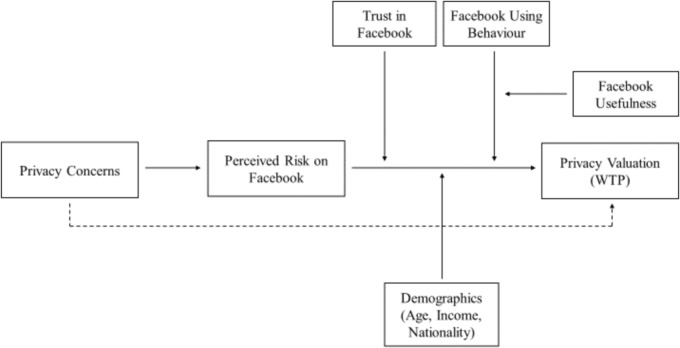
Theoretical model underlying privacy valuations.

To unravel the psychological mechanisms underlying privacy valuations on social networking services, a theoretical model will be tested. The present model is developed based on previously suggested models by Wilson and Valacich (2012) and Malhotra et al. (2004). The theoretical model presented here outlines the expected relationships between the psychological variables in predicting Internet users’ privacy valuations on social networking services (see **Figure 4**). The modeled psychological variables are selected based on previous research demonstrating their relevance in the context of information privacy. Where necessary, the psychological measures are adapted to suit the context of Facebook.

The present model proposes that perceived risk on Facebook mediates the relationship between privacy concerns (see also Malhotra et al., 2004) in predicting valuation of privacy, and that this relationship is further moderated by trust in Facebook and its Data Policies (adapted from Milne and Culnan, 2004) as well as by the level of Facebook use (adapted from Jenkins-Guarnieri et al., 2013). More specifically, we propose that high levels of privacy concerns predict high willingness-to-pay for privacy, mediated through increased privacy-related risk perception on Facebook. Additionally, the valuation of privacy is expected to depend on Facebook members’ current Facebook use or non-members’ perceived usefulness of Facebook, respectively (adapted from Rauniar et al., 2014). Among frequent Facebook users, those with greater privacy concerns are expected to express greater willingness-to-pay for privacy on Facebook than those with lower privacy concerns. Among non-members of Facebook, those with strong privacy concerns and perceptions of Facebook’s usefulness are expected to express higher willingness-to-pay for privacy than those non-members who do not perceive Facebook as useful. The rationale behind this is that privacy-concerned people who perceive Facebook as useful but are not member of the network, may abstain due to their privacy concerns, rather than due to lacking benefits from Facebook membership, and may thus be more likely to pay for privacy on Facebook. In addition to these psychological characteristics, socio-demographic information and the psychological characteristics social norms and comparative optimism will also be assessed, as these may have additional explanatory power beyond the primary variables included in the model. The psychological characteristics and socio-demographic information that are expected to explain participants’ privacy valuations are explained in more detail in the next section (see Section Psychological Characteristics).

### Psychological Characteristics

#### Privacy Concerns

The IUIPC scale developed by Malhotra et al. (2004) is a widely used measure of privacy concerns consisting of 10 items. The items (e.g., *“It usually bothers me when online companies ask me for personal information”*) assess the three privacy dimensions data collection, data control, and awareness of the company’s data practices on a 7-point Likert scale from one (strongly disagree) to seven (strongly agree). All three subscales have a composite reliability score of above 0.70 and have been validated in predicting behavioral intentions and Internet users’ reactions to online privacy threats (Malhotra et al., 2004). The relationship between privacy concerns and willingness-to-pay for privacy on social networking services will be examined. It is hypothesized that high levels of privacy concerns will predict greater willingness-to-pay for privacy directly through perceived risks on Facebook as well as through moderation of further psychological characteristics.

#### Perceived Risk on Facebook

Along with the IUIPC, Malhotra et al. (2004) used and adapted the risk perception scale validated by Jarvenpaa et al. (1999). As suggested in Malhotra et al. (2004), we adapted the six risk perception items to make them specific to the context of Facebook (e.g., *“The risk that personal information submitted to Facebook could be misused is immense”*). The scale has a reliability score of Cronbach’s α = 0.70 and uses a 7-point Likert scale ranging from one (strongly disagree) to seven (strongly agree). We hypothesize perceived risk on Facebook to be the main mediator of the effect of privacy concerns on willingness-to-pay. For participants with high privacy concerns but low risk perceptions on Facebook, however, valuation of privacy is expected to be low.

#### Perceived Internet Privacy Risk and Personal Internet Interest

Two scales will be used that were developed and validated by Dinev and Hart (2006a) and measure general Internet privacy risk and interest. Perceived Internet privacy risk consists of four items (e.g., *“I am concerned that the information I submit on the Internet could be misused”*), while personal Internet interest consists of three items (e.g., *“The greater my interest to obtain a certain information or service from the Internet, the more I tend to suppress my privacy concern”*). The items are assessed on a 5-point Likert scale ranging from one (very low risks/strongly disagree) to five (very high risk/strongly agree). For both scales, Cronbach’s alpha indicates reliability above 0.66, which is the recommended cut-off score (Nunnally, 1978). Dinev and Hart (2006a) find that higher privacy risk perceptions are related to higher levels of privacy concerns and lower willingness to transact personal information on the Internet, and that higher Internet interest is related to higher willingness to transact personal information on the Internet. While perceived risk on Facebook (see Section Perceived Risk on Facebook) is included as the main mediator in the model, the more general perceived Internet privacy risk measure will be tested as potential moderator for non-members of Facebook.

#### Trust in Facebook

Trust has been described as important foundation for all economic transactions (Ben-Ner and Halldorsson, 2010) and previous research demonstrated that customers’ trust in companies and the Internet are important predictors of online disclosure and sharing behaviors (Metzger, 2004). Trust in the social networking service Facebook will be assessed via the trust in privacy notices subscale by Milne and Culnan (2004), defining trust as consumers’ willingness to accept a level of risk in the face of incomplete information and as their belief that businesses will adhere to the privacy practices they declare (see Gefen et al., 2003 for a review on the trust literature). The relationship between trust in privacy notices with perceived risk and privacy concerns has been validated in Milne and Culnan (2004). In the present study, this relates to the belief that changes in Facebook’s Data Policy can generally be trusted and the scale will be adapted to the context of Facebook. The scale consists of five items (e.g., *“I believe that the Facebook privacy statements are truthful”*), which are assessed on a 5-point Likert scale ranging from one (strongly disagree) to five (strongly agree). The scale’s Cronbach’s alpha is 0.82. Trust is hypothesized to moderate the relationship between privacy concerns and willingness-to-pay for privacy. Precisely, to invoke willingness-to-pay for privacy, participants need to generally trust Facebook and trust in Facebook’s adherence to the offered privacy enhancements.

#### Facebook Use

Facebook use will be measured only among participants who, at the time of participation in this study, are members of Facebook. Facebook use will be assessed using the social media use integration scale by Jenkins-Guarnieri et al. (2013). The validated scale consists of 10 items (e.g., *“I feel disconnected from friends when I have not logged into Facebook”*), which are assessed on a 6-point Likert scale ranging from one (strongly disagree) to six (strongly agree). The scale has a Cronbach’s alpha reliability of 0.91 and assesses social integration in and emotional connectedness to Facebook. It is hypothesized that frequent Facebook use will moderate the effect of privacy concerns through risk perceptions on participants’ willingness-to-pay. Precisely, frequent Facebook users with strong privacy concerns are assumed to indicate greater willingness-to-pay.

#### Perceived Usefulness of Facebook

Perceived usefulness of Facebook will be assessed only in participants who, at the time of participation in this study, are non-members of Facebook. The perceived usefulness scale from the revised social media technology acceptance model (TAM) by Rauniar et al. (2014) will be administered and adapted to the context of Facebook. The scale has been validated by Rauniar and colleagues and consists of five items (e.g., *“Using Facebook makes it easier to stay informed with my friends and family”*), which are assessed on a 5-point Likert scale ranging from one (strongly disagree) to five (strongly agree). The scale has a composite reliability score of above 0.70. We hypothesize that perceived usefulness of Facebook will moderate the relationship between privacy concerns and willingness-to-pay for non-members of Facebook. Precisely, we expect that when non-members of Facebook with high privacy concerns and risk perceptions still consider the usefulness of Facebook to be high, they could be willing to use a version of Facebook that protects their data and therefore indicate a higher willingness-to-pay.

#### Socio-Demographic Information

Previous research showed that socio-demographic factors, such as age and gender, influence Internet users’ valuation of personal data and privacy (e.g., Krasnova et al., 2009). Therefore, socio-demographic information will be assessed, including gender, age, level of education, employment status, type of work, socioeconomic status, country of residence, and nationality. Socioeconomic status is predicted to have an influence on willingness-to-pay, as economic status (e.g., income) impacts people’s overall readiness to pay a certain financial amount for the usage of a service or a product (Onwujekwe et al., 2009). We assume that socio-demographic information will influence the relationship between privacy concerns and willingness-to-pay for privacy on social networking services and control for these influences in our model.

#### Social Norms

Social norms are a strong predictor of human offline behaviors (Cialdini and Trost, 1998) and have been shown to be a significant antecedent of adopting online behaviors too (Chiasson and Lovato, 2001; Spottswood and Hancock, 2017). We will employ the questionnaire developed by Charng et al. (1988) to assess perceptions of social online norms and adapt the questionnaire to the context of Facebook. The questionnaire was validated for online use and has a reliability of Cronbach’s alpha of 0.86 (Choi and Chung, 2013). The five items (e.g., *“Many of the people that I know expect me to continuously use Facebook”*) are assessed on a 7-point Likert scale ranging from one (strongly disagree) to seven (strongly agree). We hypothesize that perceived social norms positively correlate with perceived usefulness of Facebook in non-members and with Facebook use in current Facebook users. Hence, social norms could further moderate the impact of privacy concerns on willingness-to-pay. If confirmed in the analysis, this variable may be included in the theoretical model.

#### Comparative Optimism

Participants’ comparative optimism in the online context will be assessed using the approach by Baek et al. (2014). This approach relies on the indirect method (Harris et al., 2000) to assess participants’ likelihood estimation of experiencing a certain event as compared to others experiencing the same event. In two separate items, participants make judgments about their perceived personal and target group risk (i.e., *“How likely are you [target group] to fall victim to improper use of online information?”*). Both items will be assessed on a 5-point Likert scale ranging from one (least likely) to five (most likely). It is expected that participants who underestimate their own risk to fall victim to improper use of online information, as compared to others, have lower privacy concerns and risk perceptions, which may result in lower willingness-to-pay for privacy. Similar to social norms, we will test the relevance of this variable for the model.

## Stepwise Procedures

The present experiment will be administered online using the web-based survey tool Qualtrics that allows designing, running, and collecting data through online experiments and surveys. The stepwise procedures of the experiment are as follows: After informed consent is given, participants will first answer a baseline measure that assesses if participants would be willing to pay for the current, free-of-charge version of Facebook. Afterward, participants will be presented a short vignette describing a scenario in which Facebook may consider developing premium versions of their service that would offer enhanced privacy for users in return for a monthly fee. In the first part of the online experiment, four hypothetical, privacy-enhanced premium versions of Facebook are presented consecutively and participants indicate their willingness-to-pay for each of the premium versions using the four questions of the PSM and the additional overall willingness-to-pay item (see Section Willingness-to-Pay Measure). Each privacy-enhanced version of Facebook is contrasted with the conventional, free-of-charge version of Facebook to facilitate comparability and increase participants’ understanding of the enhancements of the premium versions. To control for order effects, the three privacy-enhanced premium versions of Facebook (i.e., data collection, data control, and third party sharing) will be presented in randomized order. The fourth full-design premium version, which combines all three privacy enhancements in one version, will be presented last.

The second part of the study will assess several psychological characteristics (see Section Psychological Characteristics) to test the proposed theoretical model (see Section Theoretical Model) that specifies the psychological mechanisms underlying Internet users’ privacy valuations. The items of each scale will be presented in randomized order. Short control questions will be included in the online survey to ensure participants understand the privacy enhancements in the premium versions and to assess for how useful, credible, and technologically feasible these are rated. Two more general items will control whether participants answer the online study truthfully (e.g., *“In general, I answered all of the questions seriously”*). Lastly, socio-demographic information will be assessed. Once the survey is completed, participants will be thanked and further debriefed about the topic and purpose of the present study and those interested can read more about privacy and how to protect their online data. Those participants wishing to enter the prize draw will be invited to follow a link to a separate survey where they can enter their email addresses. This way participants’ anonymity will be preserved and linking survey responses to identifiable information will be avoided.

## Proposed Analysis

**FIGURE 5:**
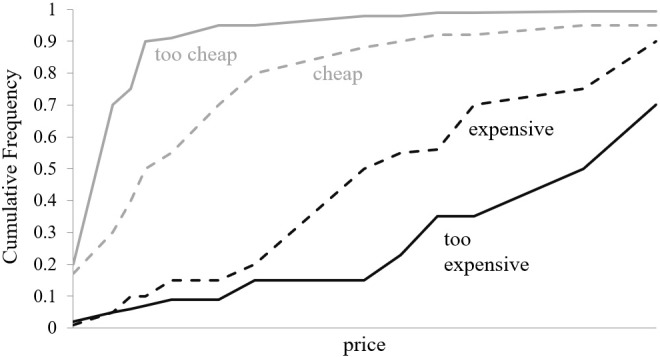
Cumulative frequencies of the questions of the PSM.

In the first step, a cumulative frequency will be calculated for each of the enhanced privacy aspects captured in the hypothetical premium versions of Facebook (**Figure 5**).

**FIGURE 6:**
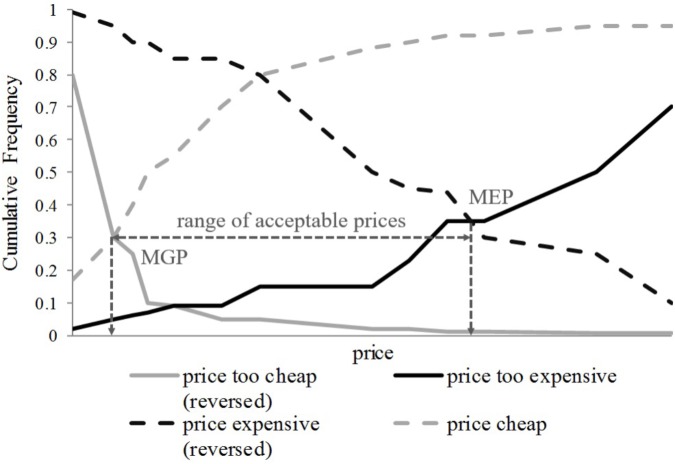
Range of acceptable prices.

In a second step, the range of acceptable prices that each participant is willing to pay for the different privacy-enhanced premium versions will be determined. The range of acceptable prices is defined by its endpoints marginal cheapness and marginal expensiveness (van Westendorp, 1976). Marginal cheapness is determined by the point where the cumulative frequencies of “too cheap” prices (reversed) and “cheap” prices intersect (MGP in **Figure 6**). In contrast, the point of marginal expensiveness is determined by the intersection of the cumulative frequencies of “too expensive” prices (reversed) and “expensive” prices (MEP in **Figure 6**).

In a third step, we will follow the approach by Lipovetsky (2006) who proposes that the four questions of the PSM and their corresponding cumulative distributions split the price continuum into five price perception intervals. These five price perception intervals are *too cheap, bargain, acceptable price, premium*, and *too expensive*. Thus, instead of the four thresholds of the questions of the PSM (**Figure 5**), five price ranges will be considered that are defined as discrete states with a continuous price variable and modeled as ordinal logistic regressions.

**FIGURE 7:**
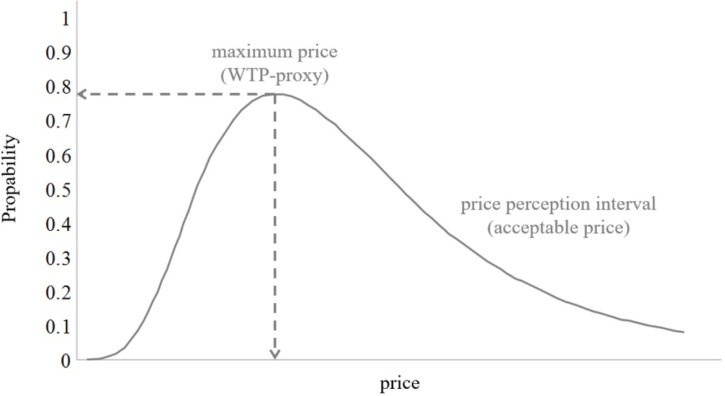
Price sensitivity for the acceptable price perception interval.

Following this model, the logistic cumulative probabilities for each price threshold will be determined and the appropriate thresholds for the particular model will be subtracted (i.e., for the acceptable price model the expensive price threshold is subtracted from the cheap price threshold). This procedure leads to smooth regression lines and allows determining the maximum of a specific price perception range. These maxima will be used as a proxy for participants’ willingness-to-pay (WTP in **Figure 7**).

Ordinal logistic regression models will be applied to test for statistical differences between participants’ willingness-to-pay for the different privacy aspects captured in the hypothetical premium versions of Facebook. Furthermore, the regression models can be extended to multiple predictors (e.g., privacy concerns and socio-demographic characteristics), since we hypothesize that psychological characteristics influence participants’ propensity to pay for the privacy enhancements. Together with the range of acceptable prices, the proxies will be used to test for intra-individual and inter-individual differences between willingness-to-pay for the four privacy-enhanced premium versions of Facebook. In addition, repeated-measure ANOVAs will be calculated for participants’ willingness-to-pay for the four different premium versions of Facebook, using the participant answers on the overall valuation measure (i.e., *“Overall, how much would be willing to pay for this premium version of Facebook?”*) as dependent variable. Where applicable, *post hoc* tests will be employed to determine the specific group differences. Data analysis will be conducted in R studio (R Core Team, 2017) and the conventional significance level of α = 0.05 will apply to all analyses.

With respect to the theoretical model (see **Figure 4**, Section Theoretical Model), we follow previous approaches (Malhotra et al., 2004; Schreiner and Hess, 2015) and assume linear relationships between the indicated psychological variables (see Section Psychological Characteristics), which will be statistically tested using structural equation modeling to identify the path coefficients. As outcome variable in the tested model, participants’ overall willingness-to-pay for the hypothetical, full-design premium version of Facebook will be used.

## Anticipated Results

In the proposed experiment, Internet users’ valuation of different privacy aspects will be investigated in the context of social networking services. Four hypothetical, privacy-enhanced premium versions of Facebook will be developed, each offering the enhancement of one specific privacy aspect, namely data collection, data control, and third party sharing. A fourth version incorporates all three privacy enhancements. Valuation of privacy will be quantified using willingness-to-pay. The main aims of the experiment are to identify differences in the valuation of the three privacy aspects as well as to unravel the psychological mechanisms underlying these valuations.

For the purpose of the study, the PSM will be employed to measure willingness-to-pay for the premium versions of Facebook. The PSM allows estimating acceptable price ranges for each of the examined privacy aspects. Ordinal logistic regression as well as ANOVAs and according *post hoc* testing will be employed to investigate within-subject valuations of the three privacy aspects (i.e., data collection, data control, and third party sharing). In a second analysis step, the proposed theoretical model encompassing relevant psychological characteristics will be tested in order to unravel the psychological mechanisms underlying valuations of privacy. We expect overall willingness-to-pay (i.e., for the full-design premium version of Facebook) to be explained by privacy concerns, mediated by the perceived risk on Facebook, as well as by several moderating variables (see Sections Theoretical Model and Psychological Characteristics).

The results from this study will be a valuable contribution to the existing literature on information privacy. Most of the previous research has treated privacy as a one-dimensional construct and, thus, has not addressed consumer valuation of different aspects of privacy. Also, previous studies have largely disregarded non-members of social networking services, who constitute a large subsample that could be attracted to join social networking services, if these offered users enhanced privacy. The findings will, hence, complement several previous studies that examined the privacy paradox and valuation of privacy (e.g., Tsai et al., 2011) by offering a more detailed examination of the valuation of different privacy aspects, while also including non-members of certain services and products in this examination. Moreover, the findings will provide insights into the psychological mechanisms underlying these valuations. In comparison to Schreiner and Hess (2015), for example, who explained willingness-to-pay for privacy-enhanced premium services using the theory of planned behavior, the model proposed in this study emphasizes risk perceptions as a mediator for the effect of privacy concerns on willingness-to-pay for privacy on social networking services. It thereby focuses less on the valuations of the premium version itself, and rather serves to explain the individual differences in online privacy valuations. Furthermore, Schreiner and Hess did not find a link between perceived Internet risk and willingness-to-pay for privacy-enhanced premium services. We suggest that the use of a general risk perception measure, rather than a Facebook-specific measure, could likely account for the unidentified link between these two related constructs. Therefore, in the present study, we will use a risk perception measure adapted specific to the context of Facebook. Besides the novel scope and the adapted constellation of the psychological factors in our proposed model, the present model also adds a cross-cultural dimension by sampling participants internationally and across cultures. Previous studies often collected data in only one country (e.g., Schreiner et al., 2013) or were predominantly relying on student populations (e.g., Krasnova et al., 2009).

Beyond the scientific contributions, the findings from the present research have considerable practical relevance, particularly in light of recent events such as the Cambridge Analytica Scandal (Revell, 2018) and the data protection laws that came into effect in the European Union in May 2018 (i.e., General Data Protection Regulation [GDPR]; Regulation (EU) 2016/679, 2017). Alternative business models may receive greater attention, as these could balance the asymmetric relationship between consumers and businesses and offer Internet users new privacy functionalities (e.g., Crook, 2018). Identifying which privacy features (e.g., third party sharing) are valued most, direct suggestions for the most important privacy enhancements can be derived. This will allow providing valuable suggestions for economically sustainable privacy enhancements and urgently needed alternative business models that are beneficial to consumers, service providers, and policymakers, alike.

Despite the study’s important contributions to the existing scientific literature on information privacy and its practical relevance, there are a number of limitations that need to be addressed. First, as this study relies on a hypothetical scenario, no actual behaviors will be measured. Thus, this study only provides insights into Internet users’ valuation of privacy based on hypothetical premium versions of Facebook. Though this study uses willingness-to-pay an indicator to quantify valuation of privacy, it is a rather intentional measure and does not provide a reliable economic value that translates into actual willingness-to-pay in a real-world settings (see intention-action gap; Sheeran and Webb, 2016). Second, as privacy concerns are context-dependent (e.g., Nissenbaum, 2009), the findings from this study are not generalizable to other platforms, but are specific to Facebook. Similarly, other measures assessed in this study, such as privacy concerns or risk perceptions, differ across countries, and culture (Wildavsky and Dake, 1990; Krasnova et al., 2012; Morando et al., 2014; Eurobarometer, 2016). Therefore, we will control for this by employing an international, cross-border sampling strategy. Third, despite our attempts to reach a heterogeneous sample by recruiting internationally and advertising our study on different platforms, our sample strategy may nonetheless be affected by sample bias, such as self-selection bias. Future studies could employ panel-based recruitment in order to reduce self-selection bias. Lastly, the presentation of the privacy policies will likely have an influence on users’ willingness-to-pay. Privacy policies are usually far from the brevity and level of user-friendliness offered in this experiment. Future studies could more closely investigate the influence of presentation of such policies to suggest more user-friendly alternatives and test willingness-to-pay in real-world setting using actual premium versions.

## Nomenclatures

IUIPC, the ‘Internet User’s Information Privacy Concerns’ is an instrument which measures the perception of acceptability of personal information collection practices; PSM, the ‘Price Sensitivity Meter’ is a descriptive statistical procedure used for calculating willingness-to-pay developed by van Westendorp; MGP, the point of ’marginal cheapness’ is the intersection of the reversed ‘too cheap’ curve with the ’cheap’ curve, defined by van Westendorp in his price sensitivity meter; MEP, the point of ’marginal expensiveness’ is the intersection of the reversed ’expensive’ curve with the ’too expensive’ curve, defined by van Westendorp in his price sensitivity meter; WTP, ‘Willingness-to-pay’; TAM, the revised social media ‘technology acceptance model’ by Rauniar et al. (2014); ANOVA, analysis of variance is a statistical procedure used to analyze the differences among group means in a sample; GDPR, the ‘General Data Protection Regulation’ is a regulation in European law that came into effect on 25 May 2018, serving to strengthen data protection and privacy for all individuals within the European Union and the European Economic Area.

## Ethics Statement

The proposed research was approved by the ethics committee of the Department of Psychology at the University of Geneva.

## Author Contributions

JM conceived the original idea for the research and provided supervision and guidance throughout. All authors made significant intellectual contributions to the study design and written protocol, were involved in all steps of the process, and approved the final version for publication.

## Conflict of Interest Statement

The authors declare that the research was conducted in the absence of any commercial or financial relationships that could be construed as a potential conflict of interest.

## Footnotes

1 Given that many of the participants may not be native English-speakers, participants’ English language proficiency will be assessed in a one-item self-report measure.
